# Exploration of the Biotechnological Potential of Two Newly Isolated *Haematococcus* Strains from Reunion Island for the Production of Natural Astaxanthin

**DOI:** 10.3390/foods13223681

**Published:** 2024-11-19

**Authors:** Samuel Jannel, Yanis Caro, Marc Bermudes, Thomas Petit

**Affiliations:** 1 Laboratoire de Chimie et de Biotechnologie des Produits Naturels—ChemBioPro (EA2212), Université de La Réunion, 15 Avenue René Cassin, FR-97490 Sainte-Clotilde, La Réunion, France; 2 Green Mascareignes Technologies SAS, 2 rue Maxime Rivière, FR-97490 Sainte-Clotilde, La Réunion, France; 3 Département HSE, IUT de La Réunion, 40 Avenue de Soweto, FR-97410 Saint-Pierre, La Réunion, France

**Keywords:** *Haematococcus*, astaxanthin, biodiversity, Reunion Island, photobioreactors, biomass and metabolites yields

## Abstract

*Haematococcus lacustris* is a freshwater green microalgae species able to produce and accumulate astaxanthin in response to environmental stresses such as high light and nutrient deprivation. Astaxanthin is a xanthophyll carotenoid of growing economic interest due to its numerous biological activities, notably its strong antioxidant properties, which can be valued in the fields of nutrition, health, feed and aquaculture. The present study aims at evaluating the capacity of two newly isolated *Haematococcus* strains from the biodiversity of Reunion Island, to be cultivated in a photobioreactor and to produce astaxanthin. The results showed that both strains were able to grow in various nutritive media and to produce and accumulate astaxanthin in response to stresses, mainly in the form of astaxanthin monoesters, which represented up to 2% of the dry biomass weight and which were mostly composed of linoleic and linolenic acids. In fed-batch cultures using 3 L benchtop photobioreactors, the concentrations of biomass enriched in astaxanthin reached up to 3 g L^−1^ (dry weight) with biomass productivities of 0.04 and 0.02 g L^−1^ d^−1^ based on the durations of the vegetative stage and of the entire culture, respectively. In these cultures, the astaxanthin productivities were found to reach on average around 0.25 mg L^−1^ d^−1^. Although these results were relatively low compared to the literature, the possibility of improving growth conditions in order to improve biomass and astaxanthin yields, to guarantee economic viability for cultivation at a commercial scale, was further discussed.

## 1. Introduction

Astaxanthin (3,3′-dihydroxy-β,β-carotene-4,4′-dione) is a xanthophyll carotenoid which presents numerous biological activities and which is gaining stronger economic interest. Indeed, many in vitro and in vivo studies highlighted its strong antioxidant properties, that were confirmed by a number of clinical studies which showed the benefits for human health. These interesting properties are probably linked to its unique chemical structure, which gives it the capacity to insert itself into cell membranes, particularly those of mitochondria [1]. Many other biological activities, such as those that are anti-inflammatory [2], have been reported in the literature [3,4]. Based on these properties, astaxanthin could represent a potentially great preventive agent against many chronic pathologies, such as metabolic diseases including diabetes and its many negative health consequences [5].

Natural astaxanthin can be primarily produced by some yeasts, bacteria, microalgae, and flowering plants, and is found in some crustaceans, fishes, and birds. For example, the yeast *Xanthophyllomyces dendrorhous* (although known as *Phaffia rhodozyma*) can accumulate astaxanthin up to 0.4 mg g^−1^ of the total dry weight (dw) in wild-type strains and up to 5 mg g^−1^ after mutagenesis [6]. In another study, the astaxanthin yield in the heterotrophic cultivation of a mutant could reach 420 mg L^−1^ [7]. In the bacteria *Paracoccus* sp., astaxanthin can represent more than 0.8 mg g^−1^ dw [8]. It was shown that the 0.9 mg L^−1^ astaxanthin achieved during the heterotrophic cultivation of wild strains could be improved by mutagenesis to reach up to 16 mg L^−1^ [9]. The microalga *Chromochloris zofingiensis* (*Chlorella zofingiensis*) was also capable of accumulating astaxanthin up to 5.5 mg g^−1^ [10]. Because of the high cell density that can be obtained during the heterotrophic cultivation of this species, astaxanthin yield can reach 73 mg L^−1^ with an astaxanthin productivity of more than 5 mg L^−1^ d^−1^ [11], which is eight times higher than photoautotrophic conditions [12].

However, astaxanthin can also be artificially synthesized, and this form is predominant in the global astaxanthin market, particularly intended for animal feed and aquaculture [13]. Indeed, this market was estimated in 2023 to reach more than USD 850 million [14]**,** while that of natural astaxanthin was valued at around USD 116 million when estimated for 2022. However, in response to consumer demand, it is expected that the natural astaxanthin market will significantly grow in the coming years, with an estimated compound annual growth rate (CAGR) of more than 8%, to reach almost USD 250 million in 2032 [15]. Via the consumption of crustaceans and fishes, especially salmonids, astaxanthin is already present in the human regular food diet. However, the use of astaxanthin in human nutrition is restricted to food supplements and primarily concerns the natural form derived from the microalgae *Haematococcus lacustris* [13,16].

*H. lacustris* (Girod-Chantrans) Rostafinski is a freshwater green microalgae species which belongs to the family Haematococcaceae, in the order Chlamydomonadales and the class Chlorophyceae [17]. Nowadays, this species is considered one of the best sources of natural astaxanthin according to some studies, which claimed that its content can reach up to 4% dw in optimized conditions in photoautotrophic cultivation [18,19,20]. In a previous study, two microalgal strains very close morphologically and genetically to *H. lacustris* were isolated from the biodiversity of Reunion Island and identified as belonging to the genus *Haematococcus* [21].

Reunion Island is a French overseas department located in the Indian Ocean near Madagascar and Mauritius. This mountainous and volcanic island, presenting a wide variety of climatic conditions which contribute to its unique biodiversity, is recognized as one of the 34 global biodiversity hotspots worldwide [22]. On the one hand, at the local, national, and European levels, there is a strong political and societal expectation to make the territory a hub of solutions to strengthen the resilience of the island and/or other tropical regions by inventing solutions that contribute to reducing their dependencies and vulnerabilities, as well as meeting the needs of foreign markets. In this manner, through its Smart Specialization Strategy for Social and Sustainable development (S5), the territory displays its desire to promote the “green economy”, in particular through the valorization of natural products from terrestrial and marine biodiversity in domains such as food, nutrition, health, and cosmetics [23]. On the other hand, the prevalence of metabolic diseases, particularly type 2 diabetes, is important in Reunion Island, much higher than in mainland France, and these pathologies became a regional health priority for the territory [24].

The aim of this present study was to evaluate the ability of the two locally isolated *Haematococcus* strains to grow in laboratory scale cultivation and to accumulate astaxanthin. The results should allow us to assess the opportunity to produce astaxanthin on Reunion Island from the culture of these strains and thus contribute to providing solutions to local economic and public health issues.

## 2. Materials and Methods

### 2.1. Chemicals and Reagents

All mineral components which composed nutritive media were purchased from Carlo Erba Reagents (Val-de-Reuil, France) as dichloromethane, methanol, acetone, and methyl tert-butyl ether (MTBE), which were of analytical grade. HPLC-grade MTBE, ammonium acetate, and 2-Tert-butyl hydroquinone (TBHQ) (purity 97%), as well as all-*trans*-astaxanthin (purity ≥ 97%) and all-*trans*-canthaxanthin (purity ≥ 95%) analytical standards, were obtained from Merck (Darmstadt, Germany). β,β-carotene (purity ≥ 95%), cryptoxanthin (purity ≥ 95%), lutein (purity ≥ 92%), and zeaxanthin (purity ≥ 95%) standards were supplied by Extrasynthese (Genay, France), while the USP-grade reference extract of astaxanthin esters from *H. lacustris* was purchased from Cluzeau Info Labo (Sainte-Foy-la-Grande, France).

### 2.2. Microalgal Strains

**Figure 1:**
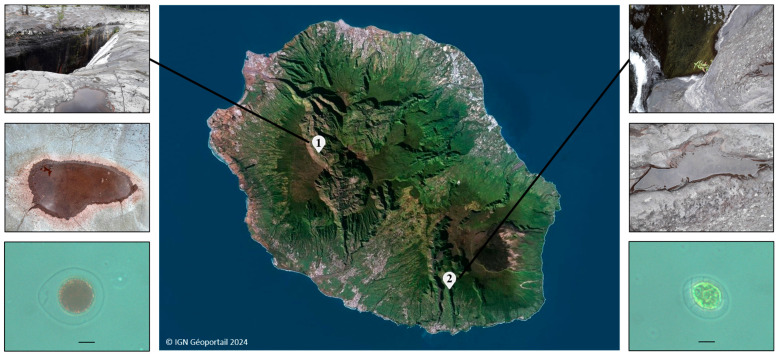
A map of Reunion Island, photographs of sample collection locations concerning the *Haematococcus* strains isolated during our previous study, and microphotographs of these strains: (1) Trois Roches waterfall, Mafate, for the strain 3RB (**left**); (2) Bassin le Jar, Langevin river, for the strain Lang2 (**right**). Scale bar 10 µm.

*Haematococcus* strains were monoclonally isolated from freshwater samples collected in different locations across Reunion Island during our previous study [21]. As a reminder, the *Haematococcus* strain Lang2, isolated on 23 December 2021, came from samples which consisted of water and sediments collected from a puddle near the Langevin river, Saint-Joseph, on 19 November 2020, at an approximate altitude of 500 m (coordinates: 21°18′48″ S, 55°38′29″ E). The *Haematococcus* strain 3RB, isolated on 3 June 2022, came from a red puddle near the Trois Roches waterfall, in the cirque of Mafate, on 28 May 2022, at an altitude of around 1200 m (coordinates: 21°05′22″ S, 55°24′40″ E) (Figure 1). These samples were subject to a request for authorization to collect them from Reunion Island National Park (Decree No. DIR-I-2024-179).

Molecular and phylogenetic analyses were previously performed on sequences of the 18S rRNA and *rbc*L genes to complete morphological characterization for taxonomic classification. The results confirmed that these strains belong to the *Haematococcus* genus, and may belong to the *H. lacustris* species, but each identification presented a relative genetic variability with this species and between the strains themselves [21].

### 2.3. Algal Cultivation, Carotenogenesis Induction and Biomass Preparation

Isolated *Haematococcus* strains were cultivated sterilely under artificial light, firstly in batch mode in growing volumes of liquid nutrient medium in culture flasks, glass flasks, and 2 L Duran bioreactors (Schott AG, Mayence, Germany), then in fed-batch mode in 3 L FMT 150/3000 benchtop photobioreactors (Photon Systems Instruments, Drásov, Czech Republic). To assess the effects on cell growth of different medium recipes and different nitrogen and phosphorus concentrations, the strain 3RB was grown in 25 mL culture flasks in Bold’s Basal Medium (BBM) [25], in BBM diluted twice (BBM/2), in BBM with a triple nitrogen concentration (3N-BBM), or the modified Blue-Green 11 medium [26], without Na_2_SiO_3_·9H_2_O and with nitrate concentrations of 0.5, 1.0, or 1.5 g L^−1^. Cultures were incubated in a climatic chamber equipped with white LED tubes at 20 °C and under a moderate irradiance (40–50 µmol photons m^−2^ s^−1^) to avoid inducing highlight stress and photoinhibition and with a 14:10 h light–dark cycles. Cell densities were frequently estimated by cell counts (see below). After up-scaling, the strain Lang2 was grown in 3 L benchtop photobioreactors equipped with a cool-white LED panel which, according to the manufacturer, emits at wavelengths between 400 and 700 nm with maximum peak values at 450 nm in the range from 540 to 590 nm. For the vegetative growth stage, these cultures were incubated in fed-batch mode at 20 °C with the BBM/2 medium and under continuous illumination of growing irradiances from 20 to 100 µmol photons m^−2^ s^−1^, and cell densities were frequently estimated by cell counts (see below). For each culture, irradiance was gradually increased as a function of cell density to ensure sufficient access of the light to the cells while avoiding inducing highlight stress and photoinhibition. To assess the effect of nitrogen availability on cell growth and to avoid induction of nutritional stress, nitrate was quantified during growth in the culture medium using Quantofix test strips (Macherey-Nagel, Düren, Germany) and a NaNO_3_ solution was added when nitrate concentrations were found between 0 and 10 mg·L^−1^, so as to reach concentrations of 1.47 mM or 2.94 mM NaNO_3_, as was the case for BBM/2 and BBM media, respectively. To evaluate the effects of pH and carbon availability on cell growth, the first culture (Lang2 FMT 1) was performed without pH regulation, while for the second and the third cultures (Lang2 FMT 2 and Lang2 FMT3) pH was set to 7.6 and was controlled by an automatic supply of 100% CO_2_. To promote carotenoid biosynthesis by the microalgal cells, cultures were exposed to higher irradiances from 100 to 500 µmol photons m^−2^ s^−1^ and deprived of nitrate to generate light and nutritional stresses, respectively. To compare the effects of different irradiances on carotenoid production, a maximum irradiance of 200 µmol photons m^−2^ s^−1^ was applied to Lang2 FMT 1, 500 µmol photons m^−2^ s^−1^ to Lang2 FMT 2, and 100 µmol photons m^−2^ s^−1^ to Lang2 FMT 3. At the end of vegetative growth and carotenogenesis stages, determined when there was no more cell division and when almost all the cells were red, respectively, biomasses of cell suspensions were harvested by centrifugation in pre-weighed 50 mL conical tubes, at 3000× *g*, at room temperature and for 10 min. The pellets were rinsed twice with distilled water and stored at −80 °C for subsequent freeze-dryings, which were performed by a FreeZone 2.5 Freeze-drier (Labconco, Kansas City, MO, USA). Freeze-dried biomasses were then weighted using an analytical balance and stored for subsequent analyses.

### 2.4. Growth Analyses

To evaluate growth in vegetative phase, 1 mL samples of each culture were regularly collected (2–3 days) and fixed with Lugol’s solution (at 2% in the samples). The cell densities (Cd), expressed as the number of cells per mL, were estimated by counts on four replicates of the same samples under light microscope (MOTIC BA310, Xiamen, China) using a Malassez hemocytometer (Marienfeld Superior, Lauda-Königshofen, Germany). The results allowed us to plot the growth curves representing the cell densities in function of the cultivation time. Based on these growth curves, the following parameters were calculated: the maximum specific growth rate (µ_max_), expressed as day^−1^, which represents the number of cell divisions per day during the exponential phase of the growth curve, and the minimum doubling time (td_min_), expressed as day, which corresponds to the time required for doubling the cell density during the exponential phase:

$$\mu_{max}=\frac{\ln\left(N2/N1\right)}{\left(t2-t1\right)}$$

$${td}_{min}=\frac{ln2}{\mu_{max}}$$

where N2 and N1 were the cell densities at t2 and t1, respectively, which corresponds to days in exponential phase. Moreover, to have a more global view of the growth, the mean growth rates (µ_mean_) and the mean doubling times (td_mean_) were also calculated during the first 15 days of growth.

### 2.5. Carotenoid Extraction

In darkness, at ambient temperature and under nitrogen atmosphere, the freeze-dried biomasses (10 mg) were homogenized using a 15 mL Dounce homogenizer and pigments were extracted with 2 mL of a mixture of dichloromethane–methanol (25:75, *v*/*v*) [27]. Then, the mixture was centrifuged at 10,000× *g* for 5 min and the supernatant was collected. The extraction procedure was repeated until the pellet was colorless (at least 5 times) and supernatants were combined and evaporated to obtain a volume of 10 mL (adapted from [20]). The pigmented extracts were stored at −20 °C for subsequent saponification and/or high-performance thin layer chromatography (HPTLC), high-performance liquid chromatography (HPLC) and mass spectrometry analyses (see below). The saponification of xanthophyll esters was performed on some extracts with the addition of one volume of 0.1 M NaOH in methanol for four volumes of pigment extract and the mixture was kept overnight at 5 °C under nitrogen in darkness (adapted from [28]).

### 2.6. Carotenoid Extract Analyses

Qualitative analyses were conducted in reverse phase using a HPTLC Camag (Muttenz, Switzerland), which was composed of an Automatic TLC Sampler 4, an Automatic Developing Chamber, a TLC Visualizer 2 and a TLC Scanner 4, on 20 × 10 cm glass-packed C18 RP HPTLC silica gel plates with 0.2 mm layer thickness (Merck, Darmstadt, Germany). Prior to use, plates were pre-developed in a mixture of methanol–dichloromethane (1:1, *v*/*v*) and dried for 20 min at 100 °C in an oven. Carotenoid standards (1 mg free astaxanthin, 5 mg lutein, 5 mg zeaxanthin, 1 mg cryptoxanthin, 10 mg β,β-carotene and 2 mg canthaxanthin) were individually dissolved in acetone using volumetric flasks to obtain stock solutions at a concentration of 20 µg mL^−1^. Then, a carotenoid standard mix solution was prepared by mixing 200 µL of each stock solution into a 1.5 mL vial. A commercial reference extract of astaxanthin esters from *H. lacustris* (100 mg) was also dissolved in acetone to prepare a pigment solution at 100 µg mL^−1^. The standard mix solution and the samples were applied with a volume of 50 µL, while the reference extract from *H. lacustris* solution was applied at 20 µL. Then, the plates were developed in a mixture of methanol–acetone (1:1, *v*/*v*) which contained 0.1% TBHQ [29,30]. Finally, the developed plates were visualized under white light and UV at 366 nm, then scanned at wavelengths between 190 and 900 nm.

Qualitative and quantitative analyses were performed through HPLC in reverse-phase using a Vanquish UHPLC System (Thermo Fisher Scientific, Waltham, MA, USA) equipped with a photodiode-array detector (DAD). The separation of the pigments was performed on a Carotenoid C_30_ column (150 × 4.6 mm i.d., 3 µm) (YMC, Kyoto, Japan) connected to a guard cartridge (10 × 4.0 mm i.d., 3 µm) at 25 °C. The mobile phase consisted of methanol (A) and methyl tert-butyl ether (MTBE) (B) and the following gradient was applied: 0–8 min, 0% B; 8–14 min, 0–22% B; 14–24 min, 22% B; 24–29 min, 22–40% B; 29–45 min, 40% B. The flow rate was 1.0 mL min^−1^ and the injection volume was 20 µL. The chromatographic peaks were measured at a wavelength of 480 nm and spectra were acquired in the range from 200 to 800 nm in a 3D field mode (adapted from [31]). The dosage of astaxanthin in the extracts was performed against calibration curves plotted from the analyses of solutions of an analytical standard of all-*trans*-astaxanthin at concentrations of 5, 10, 20, 30 and 40 µg mL^−1^ in acetone made using volumetric flasks (Simax, Sázava, Czech Republic).

Liquid chromatography coupled with mass spectrometry (LC-MS) was performed using an UPLC-DAD-MS Shimatdzu (Kyoto, Japan) using the same method as that for HPLC analyses except in positive ionization mode, for which eluent A consisted of methanol with 28 mM of ammonium acetate. The electrospray ion (ESI) source was operated in positive and negative ionization modes with capillary voltages of 4.5 kV and −4.5 kV, respectively. Mass spectra were scanned in the mass range (*m*/*z*) of 100 and 900.

### 2.7. Statistical Analyses

For analyses performed in multiplicate, all data were presented as mean ± standard deviation. All cellular densities were estimated with the count of four replicates of the same sample. The outliers were identified using the Dixon and the Grubbs tests, with a significance level α of 0.05, and replaced by the sample’s average. Other means were calculated on three independent replicates (n = 3). Then, data were submitted to a one-way analysis of variance (ANOVA) to measure the statistical differences among the means. In this analysis, the normality and the homoscedasticity were analyzed using the Shapiro–Wilk and the Levine tests, respectively (α = 0.05), while a post hoc Tukey’s test was used to compare differences which were considered statistically significant when *p*-value < 0.05. All statistical analyses were performed using the XLSTAT software version 2023.3.1 (XLSTAT statistical and data analysis solution was purchased from Addinsoft and accessed from https://www.xlstat.com/fr on 12 March 2024).

## 3. Results

### 3.1. Haematococcus Strains Cultivation

**Figure 2:**
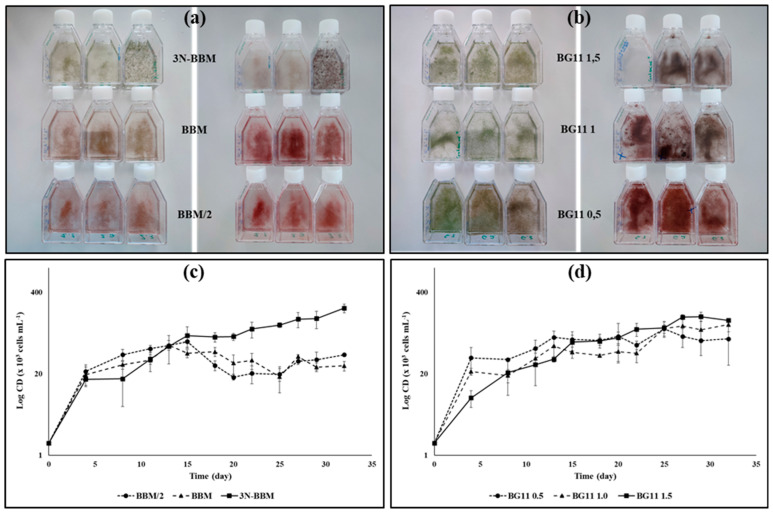
Photographs and growth characteristics of the *Haematococcus* strain 3RB, cultivated in different culture media with different nitrate concentrations: (**a**,**c**) growth in BBM based media: (**a**) after 25 days (left panel) and 56 days (right panel) of growth; (**b**,**d**) growth in BG11 based medium: (**b**) after 25 days (left panel) and 56 days (right panel) of growth; (**c**,**d**) expressed in log of cellular density in function of time. Bars represent standard deviations (n = 3).

**Figure 3:**
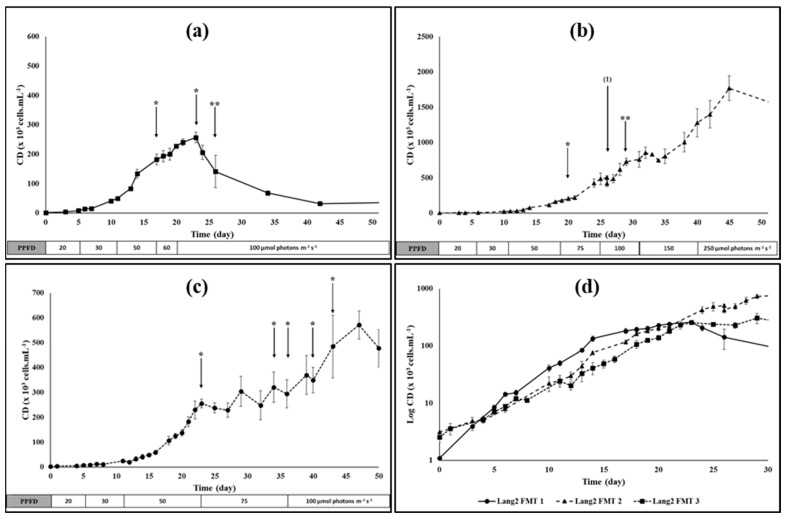
Growth curves of the *Haematococcus* strain Lang2 during the green stage of culture in 3 L benchtop photobioreactors in BBM/2 medium under increasing photosynthetically active photon flux density (PPFD): (**a**) Lang2 FMT 1 without pH control by CO_2_; (**b**) Lang2 FMT 2 with pH control by CO_2_; (**c**) Lang2 FMT 3 with pH control by CO_2_; (**d**) A comparison between the three cultures, where cell densities are expressed in a logarithmic scale. Bars represent standard deviations on four replicates of the same sample; the arrow with (1) represents a harvest of 600 mL of culture replaced by fresh medium; arrows with (*) and (**) represent the addition of 1.47 mM and 2.94 mM NaNO_3_, respectively.

The isolated Haematococcus strains 3RB and Lang2 were grown at different scales and conditions to explore their ability to be cultivated in photobioreactor. In culture flasks, the strain 3RB was found to grow in all media tested. While the best maximal growth rate (µ_max_ = 0.77 d^−1^) was obtained using the BG11 medium at 0.5 g L^−1^ of nitrate, 3N-BBM was the most suitable medium in the longer term (µ_max_ = 0.58 d^−1^, µ_mean_ = 0.26 d^−1^, Cd_max_ = 226 × 10^3^ cells mL^−1^) (Figure 2 and Table 1). The strain Lang2 was grown in a 3 L benchtop FMT photobioreactor in the medium BBM/2. The third culture of Lang2 in FMT presented the highest biomass productivities (0.04 g L^−1^ d^−1^ in dry weight (dw) in vegetative stage, 0.02 g L^−1^ d^−1^ dw based on the total duration of the culture) with a µ_max_ of 0.49 d^−1^, a µ_mean_ of 0.20 d^−1^, and a final biomass concentration of 3.1 g L^−1^ dw (Figure 3 and Table 2 and Table 3).

#### 3.1.1. Cultivation of the *Haematococcus* Strain 3RB

The *Haematococcus* strain 3RB was first tested for growth in 25 mL cultivation flasks with different media to determine which of them was most suitable for cultivating this strain. The appearance of the cultures after 25 and 56 days of cultivation was presented in Figure 2a,b, which showed that the strain was able to significantly grow on all tested media. However, it was visible that the cultures in low content nitrogen media (BBM/2, BBM, BG11 0.5) turned to a red color more quickly than those in media containing higher nitrogen content such as 3N-BBM, BG11 1.0, and BG11 1.5. This result was expected, because it is well known that nitrogen limitation induces astaxanthin production and accumulation in Haematococcus cells [32,33].

The growth kinetics of the strain 3RB were also monitored regularly during the first 32 days (Figure 2c,d), which allowed us to calculate the main growth parameters (Table 1). The results showed that the µ_max_ values were between 0.41 d^−1^ and 0.77 d^−1^, and the td_min_ values were between 0.91 day and 1.75 days. Moreover, there were statistically significant differences when comparing cultures using BG11 1.5 medium, which presented the lowest µ_max_, and consequently the highest td_min_, and those with BBM/2, BG11 0.5, and BG11 1.0 media for µ_max_, plus those with BBM concerning td_min_. In contrast, there were no statistically significant differences for all the cultures with respect to µ_mean_ and td_mean_.

The most noticeable differences between cultures were found for Cd_max_ and the time required to reach these densities. Indeed, the growth of the cultures performed in low content nitrogen (BBM/2 and BBM) significantly stopped more rapidly compared to the others, and Cd_max_ remained low. On the contrary, the cultures performed on 3N-BBM showed significantly higher Cd_max_ than other cultures, even if differences with those containing BG11 1.5 were not statistically significant. These results highlighted that nitrogen availability is an important growth-limiting factor as one of the major constituents of biomass, entering into the composition of many compounds required for many fundamental physiological functions, such as nucleic and amino acids, proteins and chlorophylls [34].

The *Haematococcus* strain 3RB was then successfully cultivated in 250 mL flasks to obtain enough biomass for pigment extractions and analyses (see below). However, for undetermined reasons, we failed in scaling-up the cultures in a 2 L bioreactor or a 3 L benchtop photoreactor. This surprising observation was confirmed on several media such as BBM/2, BBM, 3N-BBM, and BG11.

#### 3.1.2. The Cultivation of the *Haematococcus* Strain Lang2

From the first steps of isolation, it was observed that, at low cellular densities, the *Haematococcus* strain Lang2 grew better in the BBM/2 medium compared to media richer in nitrogen. Because the initial cellular densities were relatively low (from 1 × 10^3^ to 3 × 10^3^ cells·mL^−1^), this medium was chosen for cultivation of this strain in 3 L benchtop photobioreactors. Three main tests were performed, in which illumination was progressively increased together with an increase in cell density, and NaNO_3_ was punctually added when nitrogen was determined to be limiting. In the first culture (Lang2 FMT 1), CO_2_ was not supplied, and pH was consequently not regulated. On the contrary, the two other cultures were performed with automatic adding of CO_2_ and pH regulation. Another difference between cultures was that 1/5 of the volume was harvested from the culture Lang2 FMT2 after 26 days and replaced by fresh BBM/2 medium. The growth characteristics of these cultures during the green vegetative stage are presented in Figure 3, with the associated growth parameters in Table 2.

The results showed that µ_max_ and td_min_ were similar for the three experiments, with an average of 0.52 ± 0.03 d^−1^ and 1.34 ± 0.07 days, respectively. There was higher discrepancy between cultures with respect to µ_mean_ and td_mean_, which were of 0.26 ± 0.08 d^−1^ and 2.84 ± 0.75 days in average, respectively, but which reached 0.34 d^−1^ and 2.02 days, respectively, for the culture Lang2 FMT1. Overall, these results were comparable to those obtained for the strain 3RB in the same medium at a smaller scale.

The main difference between all three cultures was observed for the maximum cell densities. Indeed, in the cultures without pH regulation through a CO_2_ supply, the growth and the cell density dropped dramatically after 23 days, while they were prolonged until 45 or 47 days for the other cultures. As a consequence, the maximum cell densities were higher in the cultures supplied with CO_2_ than those without a CO_2_ supply, with a Cd_max_ that reached approximately 5.7 × 10^5^ cell mL^−1^ in the third culture and more than 1.7 × 10^6^ cell mL^−1^ in the second one.

Another observed difference was the concentration of dry biomass and the biomass yield. At the end of the green vegetative stage, 1.886 g L^−1^ of dry biomass could be harvested from the culture Lang2 FMT3, while only 0.424 g L^−1^ from Lang2 FMT1. The corresponding biomass yields of both cultures were estimated at 0.040 g L^−1^ d^−1^ dw and 0.018 g L^−1^ d^−1^ dw, respectively.

**Figure 4:**
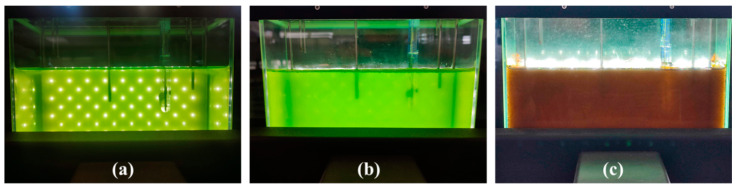
Photographs of the culture Lang2 FMT 2 in a 3 L benchtop photobioreactor in BBM/2 medium: (**a**) after 17 days; (**b**) after 25 days; (**c**) after 52 days.

The cultures were then maintained in limiting nutrient conditions and under relatively high illumination for variable duration in order to induce stress and consequently the production of secondary carotenoids, of which primarily astaxanthin. During this red stage, illumination was increased to 200 µmol photons m^−2^ s^−1^ for Lang2 FMT1, progressively to 500 µmol photons m^−2^ s^−1^ for Lang2 FMT2, while it was maintained at 100 µmol photons m^−2^ s^−1^ for Lang2 FMT3. The appearance of the culture Lang2 FMT 2 during the green vegetative stage and at the red aplanospores stage is presented in Figure 4, while the growth parameters calculated at the end of cultivation times are presented in Table 3.

At the end of the red stage, the cellular density varied between cultures in a wide range from 361.67 ± 112.59 to 1253.33 ± 277.77 × 10^3^ cells mL^−1^. Compared to the cell densities observed at the end of green stage, the cell density of the culture Lang2 FMT1 was higher (Table 2 and Table 3), probably because CO_2_ was supplied at day 85 and induced cell division. On the contrary, the cellular densities of both the cultures Lang2 FMT2 and Lang2 FMT3 were lower at the end of the red stage, probably because nutritional and/or high light stress could have induced cell death. Dry biomass concentrations were also found to be different between cultures from 1.690 to 3.104 g L^−1^, possibly because of the cultivation time (Table 3). However, despite the differences between the treatments and these results, the biomass productivities were comparable and varied from 0.019 to 0.025 g L^−1^ d^−1^ dw with an average of 0.022 ± 0.003 g dw biomass per liter and per day.

### 3.2. Chromatographic and Spectrometric Analyses

**Figure 5:**
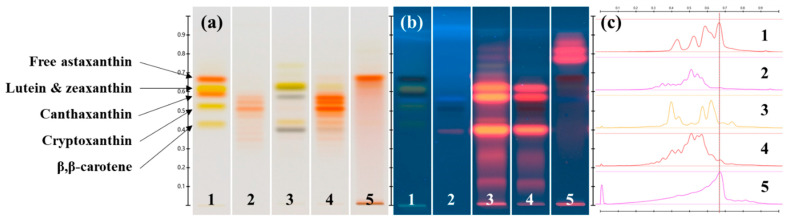
HPTLC chromatograms of the pigment extracts obtained from the isolated *Haematococcus* strain 3RB: (**a**) under transmitted white light illumination; (**b**) under reflected 366 nm light illumination; (**c**) densitogram for the transmitted white light illumination. Track 1: carotenoid standards; track 2: astaxanthin esters from *H. lacustris* reference extract; track 3: during the vegetative green stage; tracks 4 and 5: during the aplanospore red stage before and after saponification, respectively. The line on the densitogram indicated R_F_ of free astaxanthin.

**Figure 6:**
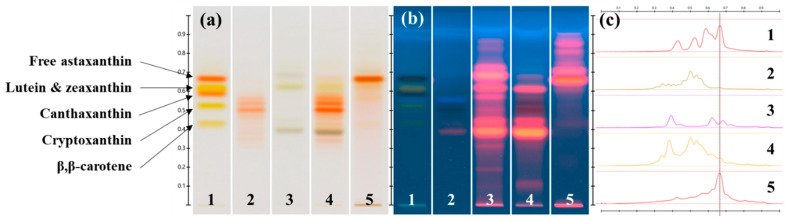
HPTLC chromatograms of the pigment extracts obtained from the isolated *Haematococcus* strain Lang2: (**a**) under transmitted white light illumination; (**b**) under reflected 366 nm light illumination; (**c**) densitogram for the transmitted white light illumination. Track 1: carotenoid standards; track 2: astaxanthin esters from the *H. lacustris* reference extract; track 3: during the vegetative green stage; tracks 4 and 5: during the aplanospore red stage before and after saponification, respectively. The line on the densitogram indicated R_F_ of free astaxanthin.

**Figure 7:**
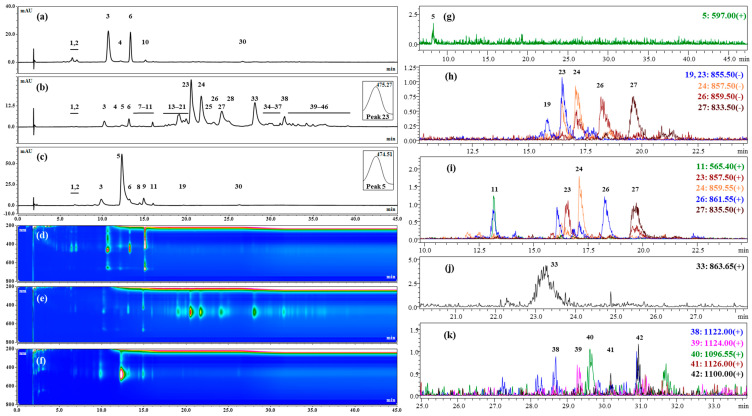
HPLC and LC-MS chromatograms of pigment extracts obtained from the *Haematococcus* strain 3RB: (**a**,**d**) in the vegetative green stage; (**b**,**e**) in the aplanospore red stage; (**c**,**f**) in the aplanospore red stage after saponification; (**a**–**f**) HPLC: (**a**–**c**) detection with DAD at 480 nm; (**d**–**f**) detection in a 3D field mode; (**g**–**k**) LC-MS: (**g**,**i**–**k**) in positive ionization mode; (**h**) in negative ionization mode.

**Figure 8:**
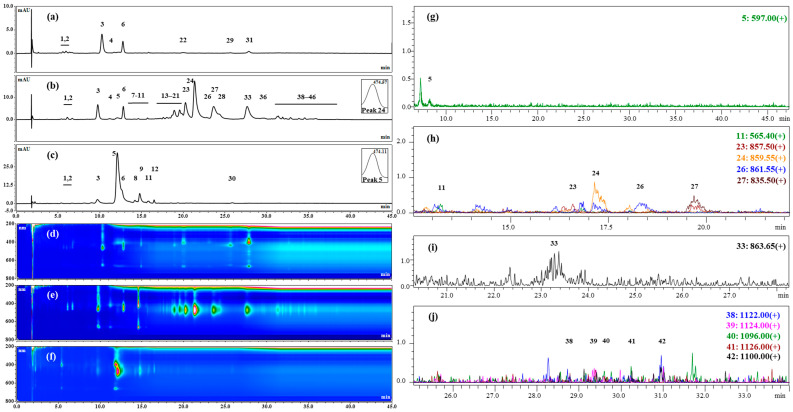
HPLC and LC-MS chromatograms of pigment extracts obtained from the *Haematococcus* strain Lang2: (**a**,**d**) in the vegetative green stage; (**b**,**e**,**g**,**h**) in the aplanospore red stage; (**c**,**f**) in the aplanospore red stage after saponification; (**a**–**f**) HPLC: (**a**–**c**) detection with DAD at 480 nm; (**d**–**f**) detection in a 3D field mode; (**g**–**j**) LC-MS in positive ionization mode.

Pigment extraction was performed from the biomasses harvested from the cultures of the strains 3RB and Lang2 in the green stage and in the red stage, and were analyzed qualitatively by HPTLC (Figure 5 and Figure 6), HPLC, and mass spectrometry (Figure 7 and Figure 8 and Table 4). The results showed high similarities in the pigment profiles of both strains and highlighted the presence of secondary carotenoids in the red biomasses, such as astaxanthin esters and, to a lesser extent, free astaxanthin and canthaxanthin, which were not detected in the green biomasses. Astaxanthin esters were mainly present in the form of monoesters and were mostly composed of stearic acid or its unsaturated derivatives. The green biomasses mainly contained chlorophylls and some primary carotenoids, such as lutein and β,β-carotene. After the saponification of the extracts, quantitative HPLC analyses showed that the red biomasses of the strain 3RB contained on average almost 1.6% *w*/*w* dw of astaxanthin and those of Lang2 had more than 1.1% *w*/*w* dw, with a global astaxanthin productivity that was around 0.25 mg L^−1^ d^−1^ dw (Table 5).

#### 3.2.1. Chemical Analyses by HPTLC of the Major Pigments of the Extracts

In more details, the HPTLC chromatograms of the pigment extracts obtained from the strains 3RB and Lang2, in comparison with those of a mixture of carotenoid standards and a commercial reference extract of astaxanthin esters from *H. lacustris*, are presented in Figure 5 and Figure 6, respectively.

Six main bands were detected on the chromatogram of the extract obtained from the green biomass of the strain 3RB, corresponding to six peaks on the associated densitogram (Figure 5a,c, track 3). Among them, three bands (retention factor (R_F_) = 0.397, 0.573 and 0.628) could correspond to chlorophylls, because their wavelengths of maximum absorbance (λ_max_) were found at around 414–427 nm and 656–669 nm, which were comparable to those of chlorophyll a [35,36,37]. The presence of these three types of chlorophylls was confirmed by the fluorescence of the bands under illumination at 366 nm (Figure 5b, track 3). The band at R_F_ = 0.441 could correspond to β,β-carotene (λ_max_ at 424 and 455 nm), while another band, overlapped to that of a chlorophyll, could represent lutein (R_F_ = 0.628; λ_max_ at 449 and 474 nm) since their R_F_ and λ_max_ were comparable to those of their respective standards. Another band (R_F_ = 0.726; λ_max_ at 405, 429 and 455 nm) corresponded to a carotenoid that remained undetermined.

The chromatogram of the extract obtained from the green biomass of the strain Lang2 shared some similarities with that of the strain 3RB, with the three bands of chlorophylls at R_F_ = 0.392, 0.577 and 0.621, and with λ_max_ at 417–427 nm and 660–667 nm (Figure 6a,c, track 3). However, under illumination at 366 nm, three other bands, significantly more intense than in 3RB, were observed at R_F_ = 0.438, 0.680, and 0.723 (Figure 6b, track 3) with λ_max_ at 407–417 and 665–668 nm, and could correspond to other chlorophylls, precursors, or degradation products. Another similarity was the presence of one band at R_F_ = 0.621, overlaid with a band of a chlorophyll, which showed λ_max_ at 449 and 467 nm and which could correspond to lutein. β,β-carotene could be present in small amounts with a band at R_F_ = 452.

Similarities were found between the extract obtained from the red biomass of the strain 3RB and the commercial reference extract of astaxanthin esters from *H. lacustris* (Figure 5a,c, tracks 2 and 4). Indeed, at least seven bands shared the same R_F_ (0.290, 0.350, 0.438, 0.485, 0.511, 0.543 and 0.568) and comparable λ_max_ (483–491 nm) with the reference extract. Moreover, a band had the same R_F_ (0.673) and λ_max_ (490 nm) than the standard of free astaxanthin. Lutein seemed to be also present at R_F_ = 0.628 with λ_max_ at 448 and 474 nm, as well as the three chlorophylls detected in the extract of the green biomass, as revealed under illumination at 366 nm (Figure 5b, track 4).

In comparison, the analysis of the extract obtained from the red biomass of the strain Lang2 revealed the presence of five bands which could correspond to astaxanthin esters (R_F_ = 0.339, 0.473, 0.501, 0.537, and 0.564; λ_max_ at 483–493 nm), as well as a light band which matched with free astaxanthin (R_F_ = 0.671; λ_max_ at 488 nm) (Figure 6a,c, track 4). Lutein could also be present with a band at R_F_ = 0.623 with λ_max_ at 451 and 475 nm, and, to a lesser extent, β,β-carotene at R_F_ = 0.425 with λ_max_ at 451 nm. Illumination at 366 nm also revealed that three chlorophylls were still present at R_F_ = 0.383, 0.425, and 0.610 (Figure 6b, track 4).

After saponification, the chromatograms of the extracts obtained from the red biomasses of the strains 3RB and Lang2 presented an intense band (R_F_ = 0.668 with λ_max_ = 490 nm, and R_F_ = 0.667 with λ_max_ at 494 nm, respectively) which corresponded to free astaxanthin (Figure 5a,c and Figure 6a,c, track 5). For the strain 3RB, three other bands at R_F_ = 0.760, 0.807, and 0.848 were fluorescent under illumination at 366 nm and probably corresponded to degradation products of chlorophylls (Figure 5c, track 5). For the strain Lang2, bands with similar R_F_ were fluorescent at 366 nm, as well as two bands at R_F_ = 0.667 and 0.712, which suggested that chlorophylls were not completely degraded by the saponification step (Figure 6b, track 5). In this extract, the bands corresponding to lutein and β,β-carotene were still detectable at R_F_ = 0.624 (λ_max_ at 456 and 481 nm) and R_F_ = 0.425 (λ_max_ at 462 nm), respectively (Figure 6a,c, track 5).

#### 3.2.2. Chemical Analyses by HPLC and Mass Spectrometry of the Major Pigments of the Extracts

The pigment extracts were further analyzed qualitatively by HPLC and LC-MS. The obtained chromatograms for the extracts from the strains 3RB and Lang2 are presented in Figure 7 and Figure 8, respectively. The data resulting from these analyzes are summarized in Table 4.

HPLC analyses confirmed the presence of three chlorophylls in the pigment extracts obtained from the green biomass of 3RB (Figure 7a,d; Table 4, peaks 3, 4, 10). Peak 3 consisted of a superposition of pigments including at least a chlorophyll and an undetermined carotenoid (λ_max_ at 466 nm), revealed by its intensity at 480 nm. Lutein (peak 6) was also present in significant amounts, as well as β,β-carotene (peak 30) to a lesser extent. Two other carotenoids that remained undetermined were also found in these extracts (peaks 1 and 2). In comparison, the chromatographic profile of the extract obtained from the green biomass of Lang2 (Figure 8a,d and Table 4) presented four additional peaks of chlorophylls or derivates (peaks 18, 22, 29 and 32). Lutein (peak 6) was also present, while β,β-carotene was not detected in this extract, probably hidden by a peak of chlorophyll (peak 29). As for the strain 3RB, two undetermined carotenoids were detected in these extracts (peaks 1 and 2).

At least thirty-six peaks were detected at 480 nm for the commercial reference extract of astaxanthin esters from *H. lacustris*. Peaks which matched with lutein (peak 6 at retention time (Rt) = 12.99 min and λ_max_ at 442, 451 and 469 nm), canthaxanthin (peak 11 at Rt = 15.88 and λ_max_ at 476) were detected, as well as eight peaks which could correspond to undetermined carotenoids in the retention time range between 13.66 and 18.40 min (λ_max_ at 453–480 nm). All of these compounds accounted for approximately 4.2% of the total peak area considering all detected pigments in the extract at 480 nm. On the other hand, twenty-six detected peaks could correspond to different forms of astaxanthin (i.e., non-esterified, mono-esterified and di-esterified, with a λ_max_ range from 468 to 479 nm) which represent approximately 92.2% of the total detected peak area of the extract. Among them, twelve pigments would likely be astaxanthin monoesters (for approximately 72.7% of the total amount of astaxanthin) and thirteen astaxanthin diesters (for 27.1% of the total amount of astaxanthin).

In the extract from red biomass of the strain 3RB (Figure 7b,e and Table 4), twenty-six detected peaks matched with astaxanthin esters of the reference *H. lacustris* extract or with free astaxanthin standard. These peaks accounted for around 91.2% of the total detected peak area of the extract. As for the commercial reference extract, twelve peaks (17, 19–21, 23–28, 31 and 33) could be attributed to astaxanthin monoesters, which represented approximately 85.3% of the total amount of astaxanthin, and thirteen peaks (34–46) could be astaxanthin diesters, accounting for 13.7% of the total amount of astaxanthin. Almost 1% of the total amount of astaxanthin was composed by free astaxanthin (peak 5). Lutein (peak 6) and canthaxanthin (peak 11) were also detected and accounted for 1.81% and 0.71% of the total detected peak area of the extract, respectively. Eight peaks (1, 2, 7, 8, 13–16) were carotenoids that remained undetermined and that accounted for 2.6% of the total detected peak area. Moreover, three residual chlorophylls were still present in the extract (peaks 3, 4, 10), as well as the undetermined carotenoid combined with a chlorophyll in peak 3. LC-MS analyses showed that the main astaxanthin monoester (peak 23), which accounted for approximately 23% of the total amount of astaxanthin, was composed of linolenic acid (C18:3) and presented a mass/charge ratio (*m*/*z*) of 857.5 in positive ionization mode ([M + H]^+^) (Figure 7i and Table 4). Other detected astaxanthin monoesters were composed of linolenic (peak 19; *m*/*z* in negative ionization mode ([M − H]^−^) = 855.5), linoleic (C18:2) (peak 24; *m*/*z* [M + H]^+^ = 859.6), oleic (C18:1) (peak 26; *m*/*z* [M + H]^+^ = 861.6), palmitic (peak 27; *m*/*z* [M + H]^+^ = 835.5), and stearic (C18:0) (peak 33; *m*/*z* [M + H]^+^ = 863.7) acids (Figure 7i,j and Table 4). Among astaxanthin diesters, the peak 38 presented a *m*/*z* [M + H]^+^ of 1122.0 and was composed of C18:1/C18:3 or C18:2/C18:2, the peak 39 (*m*/*z* [M + H]^+^ = 1124.0) of C18:1/C18:2 or C18:0/C18:3, the peak 40 (*m*/*z* [M + H]^+^ = 1096.0) of C16:0/C18:3 or C16:1/C18:2, the peak 41 (*m*/*z* [M + H]^+^ = 1126.0) of C18:1/C18:1 or C18:0/C18:2 and the peak 42 (*m*/*z* [M + H]^+^ = 1100.0) of C16:0/C18:1 or C16:1/C18:0 (Figure 7k and Table 4). The presence of free astaxanthin (peak 5; *m*/*z* [M + H]^+^ = 597.0) and canthaxanthin (peak 11; *m*/*z* [M + H]^+^ = 565.4) was also confirmed in this extract by the LC-MS analyses (Figure 7g,i and Table 4).

The extract obtained from the red biomass of Lang2 presented a similar chromatographic profile (Figure 8b,e and Table 4). However, in this extract, a lower number of astaxanthin esters were detected. Indeed, ten peaks could correspond to astaxanthin monoesters (17, 19–21, 23, 25, 26–28, 33) accounting for 92.2% of the total amount of astaxanthin and ten peaks would correspond to astaxanthin diesters (36–43, 45, 46) representing only 6.4% of the total amount of astaxanthin. Free astaxanthin was also present and accounted for 1.4% of the total amount of astaxanthin. The different forms of astaxanthin represented 83.7% of the total detected peak area at 480 nm, while other carotenoids, including lutein, canthaxanthin, and different minority undetermined carotenoids, accounted for at least 8.2% of the total detected peak area. Contrary to the green biomass, only three chlorophylls were still detected in the red biomass (peaks 3, 4 and 10). LC-MS analyses revealed a similar composition of this extract with respect to astaxanthin esters compared to that of 3RB, except for their respective proportion. For example, the main monoester in this extract (peak 24), which accounted for approximately 31% of the total amount of astaxanthin, was composed of linoleic acid (Figure 8g–j and Table 4).

After the saponification of the extracts obtained from the red biomass of the strains 3RB and Lang2, the peaks corresponding to free astaxanthin represented 71.0 and 71.3% of the total detected peak area at 480 nm, respectively, while almost all peaks identified as astaxanthin esters disappeared, as expected (Figure 7c,f and Figure 8c,f and Table 4). Lutein and canthaxanthin were still present, as well as some minority undetermined carotenoids. Traces of β,β-carotene were also detected, although it was not in the extract before saponification, probably hidden by another pigment. Residual chlorophylls or products of their degradation were also detectable in the sample.

**Figure 9:**
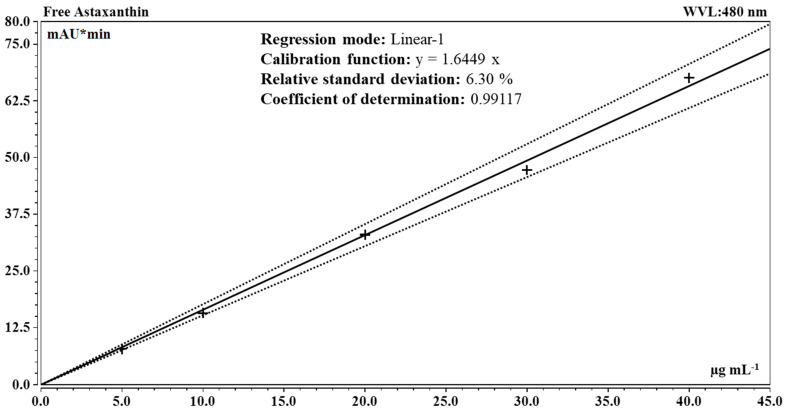
Calibration curve for free astaxanthin obtained by HPLC at 480 nm: hatched lines represent the lower and upper confidence limits at 99.5%; (+) markers represent the average of free astaxanthin analytical standard injections (n = 3–4). WVL: wavelength.

Then, quantitative analyses using HPLC were performed on saponified extracts of red biomasses harvested from different cultures of 3RB, and from the three cultures of Lang2 in 3 L benchtop photobioreactor (see above), to measure the concentration of the free astaxanthin equivalent. The calibration curve presented in Figure 9 was used for the quantification and the results are summarized in Table 5.

The analyzed biomasses of the strain 3RB showed an average astaxanthin content of 15.92 ± 3.75 mg g^−1^ dw (Table 5) with a maximum measured at 20.09 mg g^−1^ dw. In comparison, the average astaxanthin content calculated for the strain Lang2 was lower (11.35 ± 2.09 mg g^−1^ dw), even if the difference was not statistically significant. Among the different cultures of Lang2, the maximum astaxanthin content was observed at 14.94 mg g^−1^ dw in the culture FMT 1, while the highest average was found for the culture FMT 3 at 12.89 ± 0.95 mg g^−1^ dw. Based on the results in Table 3, the astaxanthin productivities in the different cultures of Lang2 varied from 0.215 mg L^−1^ d^−1^ dw for FMT 1 to 0.284 mg L^−1^ d^−1^ dw for FMT 3, with an average value of 0.251 ± 0.035 mg L^−1^ d^−1^ dw.

## 4. Discussion

The main results of this study demonstrated that the isolated *Haematococcus* strains 3RB and Lang2 were able to be cultivated in lab-scale conditions and had the capacity to produce and accumulate astaxanthin in response to nutritive and/or light stresses.

During the vegetative growth of the strain 3RB, it was shown that the use of different nutritive media with different nitrogen and phosphorus concentrations (Table 6) did not have a significant impact with respect to mean growth rates and mean doubling times, which reached around 0.26 day^−1^ and 2.70 days in average, respectively (Table 1).

Moreover, the variety of media appeared to have only a limited effect on the maximum growth rate and the minimum doubling time. Indeed, the best results (µ_max_ = 0.58–0.77 day^−1^, td_min_ = 0.91–1.20 days) were obtained with media whose nitrate concentrations were lower than or equal to 11.76 mM and with N/P ratios lower than or equal to 67 (Table 1 and Table 6). The best results (µ_max_ = 0.77 day^−1^, td_min_ = 0.91 day) were obtained with the BG11 0.5, which contained 5.88 mM of nitrogen with a N/P ratio of 33.60. On the contrary, the media with the highest nitrate concentration and N/P ratio (BG11 1.5) presented the lowest maximum growth rate (0.41 day^−1^) and, therefore, the highest doubling time (1.75 days). However, the nitrate concentration appeared to have a significant effect on the duration of vegetative growth and, consequently, on the maximum cellular density. Indeed, the cell density reached more than 225 × 10^3^ cells mL^−1^ with the use of the 3N-BBM containing 8.82 mM of nitrate, while it was significantly lower for the poorer media for which nitrate became rapidly limiting (Table 1 and Table 6). Overall, our results showed that an excessive nitrate level and/or N/P ratio can have a negative impact on cell growth kinetics at low cell densities, but apparently not in the longer term. We also confirmed that high nitrate concentrations were required to achieve high cell densities. Vegetative growth could probably be improved by using more concentrated inoculum in nitrate-rich media. To the best of our knowledge, there was no consensus in the literature about the optimal nitrogen concentration for the vegetative growth of *H. lacustris*. Indeed, even if some authors suggested that the optimal concentration is comprised of a range of 5 to 10 mM nitrogen [38], others showed that they could achieve higher cell densities at only 3 mM nitrogen [39], while one study demonstrated that the maximal biomass productivity was obtained for concentration over 10 mM nitrogen [40]. Anyway, as it was found in the present study, nitrogen concentrations below 1–1.5 mM severely limited algal growth and greatly enhanced the synthesis of astaxanthin [39,40]. A limitation in phosphorus was not highlighted in the present study, since the maximum cell densities were not significantly different between the cultures using the 3N-BBM (N/P ratio = 5) and those using the BG11 1.5 medium (N/P ratio = 100). However, a phosphate concentration around 0.5–0.6 mM was considered optimal for the vegetative growth, while a higher concentration could induce the formation of aplanospores [38] as well as a deprivation [39,41].

Relatively similar growth parameters were obtained during the vegetative growth of the strain Lang2, cultivated in a 3 L benchtop photobioreactor using the BBM diluted twice (BBM/2). Even if the average maximum growth rate for Lang2, which reached around 0.52 day^−1^, meaning an average minimum doubling time of 1.34 days, was significantly lower than for 3RB using the same medium, the average mean growth rate of 0.26 d^−1^ was almost the same (Table 1 and Table 2). For the culture of *H. lacustris,* the maximum growth rates reported in the literature could vary in the range from 0.14 to 1.30 d^−1^ [42,43,44,45], but values from 0.5 to 0.6 d^−1^ are generally observed [44,46,47,48], which was consistent with the results obtained here for the strains 3RB and Lang2. However, the punctual addition of nitrate or fresh medium and, above all, the regulation of the pH coupled with the addition of CO_2_, allowed us to reach higher cell densities up to almost 1.8 × 10^6^ cells mL^−1^ (Figure 3 and Table 2), while densities comprised of between 0.25 and 6.0 × 10^6^ cells mL^−1^ were commonly reported [39,40,45]. This yield was approximately 7 times higher compared to the culture Lang2 FMT1, achieved here without addition of CO_2_ to control pH, which reached a pH value greater than 11. This observation was in accordance with the previous studies, which demonstrated the beneficial effect of CO_2_ on vegetative growth [49,50,51], that the optimal pH was between 6.3 and 7.0 [52,53] and that a significant drop in growth was induced at pH 9 [52]. In the cultures supplemented with CO_2_, at the end of vegetative growth, the maximum dry biomass concentration obtained was about 1.9 g L^−1^ dw, with a productivity of about 0.04 g L^−1^ d^−1^ dw (Table 2). At the end of the cultivation time, after the application of nutritive and light stresses, the maximum dry biomass concentration reached 3.1 g L^−1^ dw, but the global biomass productivity fell on average to 0.02 g L^−1^ d^−1^ dw (Table 3). These maximum biomass concentrations were comparable to values reported in the literature, namely from 1.3 and 7.0 g L^−1^ dw [40,46,48,54].

Some biomass and astaxanthin productivities previously reported are summarized in Table 7, which showed that our results were in good agreement with results from Harker et al. [54], in a batch mode at a laboratory scale, and Olaizola [43], in a continuous mode at a commercial scale, using two-step culture processes as in this present study. However, these results were relatively modest compared to some more recent results, especially to the 1.9 g L^−1^ d^−1^ dw claimed by Del Río et al. [55] in a continuous mode at a laboratory scale using a one-step culture process. The low biomass productivity obtained in our study is strongly linked to the very long duration of the vegetative growth stage. This can probably be explained by initial cell densities and nitrate concentrations that were too low to allow for faster cell growth. In addition, the increase in irradiance was probably not adequate and did not allow for the optimal access of the light to cells. Finally, we aimed to obtain biomass concentrations at the end of the maximal vegetative growth phase, but the carotenogenesis phase could certainly be triggered earlier. These issues will need to be addressed in order to optimize the process in future research.

The average astaxanthin content in the red biomasses of the strain Lang2 was around 1.1% *w*/*w* dw (11.3 mg g^−1^ dw) and the average global astaxanthin productivity was approximately of 0.25 mg L^−1^ d^−1^ dw (Table 5). Similarly to biomass productivity, the astaxanthin productivity observed in our study was too low to be competitive (Table 7). As previously discussed, this was mainly due to the duration of the vegetative growth phase, but also that of the carotenogenesis phase. Our results indicated that higher irradiances (500 µmol photons m-^2^ s^−1^), combined with nitrogen deprivation, helped accelerate the carotenogenesis process compared to lower irradiances (100–200 µmol photons m^−2^ s^−1^). In this context, reducing the cellular concentration during the carotenogenesis phase, either by inducing it earlier in the vegetative growth phase or by diluting the culture, could improve cells’ exposure to light stress and potentially accelerate carotenogenesis.

Overall, these results as such are somewhat disappointing, and more specifically with respect to astaxanthin productivity, compared to other results published in the literature (Table 7), but we will redouble our efforts in order to further optimize the growth conditions to improve production yield and overall productivity, particularly with respect to initial cell density, nitrogen and light availability, and cultivation mode. Additionally, outdoor trials using natural sunlight should be conducted to assess astaxanthin productivity under real production conditions and to determine whether the climate on Reunion Island is suitable for astaxanthin production. As a comparison, the average astaxanthin content in the red biomasses of the strain 3RB was overall higher than those of Lang2, reaching almost 2% *w*/*w* dw. Unfortunately, because we were unable to cultivate the strain 3RB in photobioreactor, the astaxanthin productivity could not be calculated for this strain, as discussed previously.

From a qualitative point of view, the pigment profiles of the vegetative cells of the strains 3RB and Lang2 were found to be very similar, although more chlorophylls and/or their precursors and/or their metabolites were found for Lang2 compared to 3RB. Primary carotenoids such as lutein and, to a lesser extent, β,β-carotene, were also detected in the produced biomass. These results were well consistent with previous studies, which reported that the vegetative cells of *H. lacustris* may contain about 1.8% *w*/*w* of chlorophylls a and b and between 0.3 and 0.5% *w*/*w* of primary carotenoids [48,60]. Among them, lutein is the main carotenoid, as in this study, but neoxanthin, β,β-carotene, violaxanthin [54], antheraxanthin, and zeaxanthin [19] were also detected in this type of cells.

The carotenoid profiles of the red biomasses of 3RB and Lang2 were also similar and were composed of a majority of astaxanthin in different forms (Table 4). Canthaxanthin, another secondary carotenoid, was also detected in the red cells, as well as some minority undetermined carotenoids. Among them, some configurational isomers could be found, such as *cis*-astaxanthin, generally eluted after all-*trans* astaxanthin in reverse-phase HPLC [27,61,62] and *cis*-canthaxanthin [63]. Moreover, chlorophylls, lutein, and β,β-carotene could be still present in the red biomasses. It was previously reported that, in aplanospores, the chlorophyll content could decrease below 0.5% *w*/*w* dw [48,60] while the secondary carotenoid content increased, especially that of astaxanthin, which could represent from 80% to 95% *w*/*w* of the total carotenoid dry weight [19,54,64,65,66], but also canthaxanthin and echinenone [54,65]. It was also reported that, in this type of cells, primary carotenoids such as lutein, β,β-carotene, neoxanthin, and violaxanthin could also be present [19].

Among the different forms of astaxanthin detected in the red cells of 3RB and Lang2, astaxanthin monoesters were the more abundant form (between 85.3% and 92.2% of the total amount of astaxanthin), followed by astaxanthin diesters (from 6.4% to 13.7% of the total amount) and free astaxanthin (1.0% to 1.4% of the total amount). These results were consistent with the preliminary analyses on Lang2 [21] and with the literature [19,64], reporting that astaxanthin monoesters are the majority, then astaxanthin diesters, while free astaxanthin accounts for only 1% or 2% *w*/*w* of the total astaxanthin dry weight. The chromatographic and spectrometric analyses allowed for the detection of between 10 and 12 different astaxanthin monoesters and from 10 to 13 different astaxanthin diesters in the pigment extracts of the 3RB and Lang2 red biomasses. Once again, these results were consistent with the preliminary data reported on the strain Lang2 [21] and with the literature [61,62,63,67,68,69]. LC-MS analyses showed that, in these extracts, astaxanthin was mainly esterified with palmitic (C16:0), stearic (C18:0), oleic (C18:1), linoleic (C18:2), and linolenic (C18:3) acids, as was previously reported in the literature [61,62,63,67,68,69]. However, while the main monoesters of the commercial reference extract of *H. lacustris* were composed of C16:0 and C18:2, the main monoesters of the extracts of the strains 3RB and Lang2 were composed of C18:3 and C18:2, respectively. If the predominance of C18:2 has already been shown in some studies, in particular for commercial oleoresins [61,62], that of C18:3 seems rarer and could represent an interesting characteristic of the strain 3RB.

## 5. Conclusions

In conclusion, this study demonstrated that the two newly *Haematococcus* strains isolated from Reunion Island can be cultivated on a laboratory scale, exhibiting growth kinetics similar to those typically reported in the literature for *H. lacustris.* Under stress-inducing environmental conditions, these strains were able to produce and accumulate astaxanthin with characteristics comparable to those of commonly studied *H. lacustris* strains and commercial astaxanthin extracts from *H. lacustris*. As such, these strains show potential for valorization as dietary supplements. The biomass concentrations and astaxanthin contents achieved in this study may prove competitive, and these results are encouraging for wild strains undergoing domestication. However, the biomass and astaxanthin productivities observed with non-optimized cultivation processes are relatively modest compared to those reported in some of the literature. As a result, they are insufficient for ensuring the economic viability of large-scale cultivation. To address this, the cultivation process must be optimized to significantly reduce cultivation time and improve productivities. Future research will focus on enhancing nutrients and light availability, adjusting these factors based on biomass concentration and culture stage. Additionally, we plan to optimize environmental parameters such as pH and temperature, as well as explore exposure to combined stresses that can further stimulate astaxanthin production.

## Figures and Tables

**Table 1:** Growth parameters of the *Haematococcus* strain 3RB cultivated in different culture media with different nitrate concentrations.

Parameter	Culture Medium					
	BBM/2	BBM	3N-BBM	BG11 0.5	BG11 1.0	BG11 1.5
µ_max_ (day^−1^)	0.66 ± 0.07 ^a^	0.62 ± 0.09 ^ab^	0.58 ± 0.07 ^ab^	0.77 ± 0.11 ^a^	0.66 ± 0.03 ^a^	0.41 ± 0.08 ^b^
td_min_ (day)	1.07 ± 0.12 ^b^	1.14 ± 0.18 ^b^	1.20 ± 0.14 ^ab^	0.91 ± 0.12 ^b^	1.05 ± 0.05 ^b^	1.75 ± 0.40 ^a^
µ_mean_ (day^−1^) *	0.25 ± 0.01 ^a^	0.22 ± 0.01 ^a^	0.26 ± 0.03 ^a^	0.24 ± 0.02 ^a^	0.23 ± 0.01 ^a^	0.25 ± 0.02 ^a^
td_mean_ (day) *	2.79 ± 0.13 ^a^	3.15 ± 0.15 ^a^	2.70 ± 0.34 ^a^	2.95 ± 0.28 ^a^	3.08 ± 0.19 ^a^	2.82 ± 0.23 ^a^
Cd_max_ (×10^3^ cells mL^−1^)	66.00 ± 12.29 ^c^	58.33 ± 10.25 ^c^	225.83 ± 36.25 ^a^	105.50 ± 16.52 ^bc^	123.50 ± 29.01 ^bc^	165.33 ± 28.00 ^ab^
Time for Cd_max_ (day)	15	13	32	29	32	29

Data presented as mean ± standard deviation (n = 3). Different letters in the same line indicate statistically significant differences among groups using Tukey’s test (*p* < 0.05). * Calculated from day 0 and day 15.

**Table 2:** Growth parameters of the *Haematococcus* strain Lang2 during the green phase of cultivation in a 3 L benchtop photobioreactor in BBM/2 medium under increasing PPFD.

Parameter	Culture in 3-Liter Benchtop Photobioreactor		
	Lang2 FMT 1	Lang2 FMT 2	Lang2 FMT 3
µ_max_ (day^−1^)	0.53	0.54	0.49
td_min_ (day)	1.31	1.29	1.42
µ_mean_ (day^−1^) *	0.34	0.23	0.20
td_mean_ (day) *	2.02	3.03	3.47
Cd_max_ (×10^3^ cells mL^−1^) **	257.00 ± 17.63 ^c^	1768.75 ± 175.99 ^a^	571.25 ± 57.06 ^b^
Time for Cd_max_ (day)	23	45	47
Dry biomass (g L^−1^ dw)	0.424	Nd	1.886
Biomass productivity (g L^−1^ d^−1^ dw)	0.018	Nd	0.040

* Calculated from day 0 and day 15; ** Data presented as mean ± standard deviation on four replicates of the same sample. Different letters in the same line indicate statistically significant differences among groups using Tukey’s test (*p* < 0.05). Nd: not determined.

**Table 3:** Growth parameters of the *Haematococcus* strain Lang2 during the red phase of cultivation in a 3 L benchtop photobioreactor in BBM/2 medium.

Parameter	Culture in 3-Liter Benchtop Photobioreactor		
	Lang2 FMT 1	Lang2 FMT 2	Lang2 FMT 3
Cd (×10^3^ cells mL^−1^) *	400.75 ± 68.56 ^b^	1253.33 ± 277.77 ^a^	361.67 ± 112.59 ^b^
Dry biomass (g L^−1^)	1.918	1.690	3.104
Cultivation time (d)	101	67	139
Biomass productivity (g L^−1^ d^−1^ dw)	0.019	0.025	0.022

* Data presented as mean ± standard deviation on 4 replicates of the same sample. Different letters in the same line indicate statistically significant differences among groups using Tukey’s test (*p* < 0.05).

**Table 4:** Attempts to identify the detected pigments extracted from the *Haematococcus* strains 3RB and Lang2 red biomass using chromatographic and spectrometric data in comparison with a commercial *H. lacustris* reference extract.

Peak nb	Rt (min)	Compound	*H. lacustris* Reference Extract		3RB Green		3RB Red		3RB Red Sapo		Lang2 Green		Lang2 Red		Lang2 Red Sapo		*m*/*z* [M + H]^+^	*m*/*z* [M − H]^−^
			λmax (nm)	Rel. Peak Area ^a^	λmax (nm)	Rel. Peak Area ^a^	λmax (nm)	Rel. Peak Area ^a^	λmax (nm)	Rel. Peak Area ^a^	λmax (nm)	Rel. Peak Area ^a^	λmax (nm)	Rel. Peak Area ^a^	λmax (nm)	Rel. Peak Area ^a^		
1	6.30	UC	-	-	414, **437**, 466	4.63	**438**, 467	0.08	**439**, 467	0.08	Nd	1.86	**437**, 468	0.61	420, 446	0.42	-	-
2	6.81	UC	-	-	410, **434**, 461	2.4	410, **434**, 461	0.08	411, **435**, 461	0.27	408, **434**, 462	1.18	**434**, 461	0.03	404, 457	0.29	-	-
3	9.96	Chl + UC	-	-	**466**, 650	50.55	**468**, 650	1.91	465	9.92	**466**, 650	49.6	**466**, 652	6.46	468	4.96	-	-
4	11.70	Chl	-	-	**421**, 661	2.22	**422**, 651	0.31	468	0.76	**408**, 665	4.53	**422**, 663	0.36	-	-	-	-
5	12.20	Free Ax	479	0.21	-	-	479	0.87	475	71.03	-	-	474	1.14	**474**, 661	71.31	597.0	596.0
6	12.99	lutein	442, **469**	0.25	**442**, 469	28.19	**443**, 470	1.81	**442**, 470	2.51	442, **472**	23.25	**442**, 469	4.21	447, **470**	3.34	-	567.0
7	13.66	UC	469	0.21	-	-	**468**, 479	0.15	-	-	-	-	463	0.09	-	-	-	-
8	14.27	UC	469	0.24	-	-	471, **480**	0.16	365, **476**	2.44	-	-	476	0.12	364, **479**	2.40	-	-
9	14.84	UC	480	0.17	-	-	-	-	360, **468**	7.59	-	-	-	-	360, **468**	8.43	-	-
10	14.87	Chl	-	-	**430**, 664	2.08	**430**, 664	0.11	-	-	**429**, 664	0.51	**430**, 664	0.22	-	-	893	-
11	15.88	canthaxanthin	476	0.99	-	-	471	0.71	475	1.54	-	-	476	0.32	**443**, 470	2.07	565.4	564.0
12	16.50	UC	469, **476**	0.47	-	-	-	-	-	-	-	-	-	-	**443**, 470	1.51	-	-
13	17.35	UC	**456**, 468	0.34	-	-	458	0.38	-	-	-	-	**462**, 471	1.08	-	-	-	-
14	17.73	UC	468	0.45	-	-	464	0.54	-	-	-	-	**462**, 472	0.57	-	-	-	-
15	18.15	UC	**460**, 469	0.57	-	-	464	0.64	-	-	-	-	461	0.83	-	-	-	-
16	18.40	UC	453, **468**	0.47	-	-	464	0.61	-	-	-	-	464	0.41	-	-	-	-
17	18.89	Ax monoester	468	1.2	-	-	469	2.69	-	-	-	-	471	1.28	-	-	-	-
18	18.91	Chl	-	-	-	-	-	-	-	-	**434**, 654	0.45	-	-	-	-	-	-
19	19.22	Ax-C18:3	471	2.18	-	-	472	3.77	475	0.26	-	-	474	4.5	-	-	-	855.5
20	19.58	Ax monoester	468	2.04	-	-	473	1.79	-	-	-	-	465	3.71	-	-	-	-
21	19.81	Ax monoester	468	1.20	-	-	466	2.71	-	-	-	-	471	2.28	-	-	-	-
22	19.90	Chl	-	-	-	-	-	-	-	-	**406**, 665	2.64	-	-	-	-	-	-
23	20.39	Ax-C18:3	475	9.5	-	-	475	20.91	-	-	-	-	475	10.73	-	-	857.5	855.5
24	21.53	Ax-C18:2	474	14.93	-	-	475	15.85	-	-	-	-	475	26.2	-	-	859.6	857.5
25	22.66	Ax monoester	471	2.15	-	-	474	1.03	-	-	-	-	-	-	-	-	-	-
26	23.00	Ax-C18:1	469	1.29	-	-	471	2.4	-	-	-	-	473	1.11	-	-	861.6	859.5
27	23.89	Ax-C16:0	475	15.48	-	-	475	10.35	-	-	-	-	476	11.11	-	-	835.5	833.5
28	24.56	Ax monoester	473	5.06	-	-	474	3.19	-	-	-	-	474	3.9	-	-	-	-
29	25.67	Chl	-	-	-	-	-	-	-	-	**435**, 650	1.79	-	-	-	-	-	-
30	26.23	β,β-carotene	-	-	**450**, 472	1.17	-	-	**450**, 474	0.47	-	-	-	-	**449**, 474	0.48	-	-
31	27.52	Ax monoester	475	3.41	-	-	475	0.28	-	-	-	-	-	-	-	-	-	-
32	27.85	Chl	-	-	-	-	-	-	-	-	**407**, 665	7.02	-	-	-	-	-	-
33	27.86	Ax-C18:0	477	7.83	-	-	475	12.87	-	-	-	-	478	12.34	-	-	863.7	-
34	29.40	Ax diester	472, **478**	1.10	-	-	473	0.7	-	-	-	-	-	-	-	-	-	-
35	29.78	Ax diester	473	2.35	-	-	468	1.03	-	-	-	-	-	-	-	-	-	-
36	29.89	Ax diester	476	0.5	-	-	475	0.78	-	-	-	-	480	1.08	-	-	-	-
37	31.15	Ax diester	474	0.76	-	-	477	0.64	-	-	-	-	478	0.60	-	-	-	-
38	31.49	Ax-C18:1/C18:3 *	475	4.90	-	-	476	3.54	-	-	-	-	477	1.19	-	-	1122.0	-
39	32.03	Ax-C18:1/C18:2 *	475	1.83	-	-	476	0.87	-	-	-	-	476	0.54	-	-	1124.0	-
40	32.42	Ax-C16:0/C18:3 *	475	2.58	-	-	476	0.72	-	-	-	-	479	0.22	-	-	1096.6	-
41	33.02	Ax-C18:1/C18:1 *	475	2.74	-	-	469	0.72	-	-	-	-	468, **477**	0.51	-	-	1126.0	-
42	34.02	Ax-C16:0/C18:1 *	476	2.76	-	-	477	0.86	-	-	-	-	478	0.30	-	-	1100.0	-
43	34.75	Ax diester	475	1.1	-	-	478	0.42	-	-	-	-	476	0.42	-	-	-	-
44	35.90	Ax diester	469	1.16	-	-	479	1.00	-	-	-	-	-	-	-	-	-	-
45	36.12	Ax diester	473	2.41	-	-	478	0.98	-	-	-	-	479	0.40	-	-	-	-
46	38.78	Ax diester	477	0.55	-	-	479	0.26	-	-	-	-	480	0.12	-	-	-	-
Total astaxanthin ^ac^				92.2		0.0		91.2		71.03		0.0		83.7		71.3		
Free astaxanthin ^bc^				0.2				1.0		99.6				1.4		100.0		
Astaxanthin monoesters ^bc^				72.7				85.3		0.4				92.2				
Astaxanthin diesters ^bc^				27.1				13.7		0.0				6.4				

Nb: number; Rt: retention time; Rel.: relative; Nd: not determined; Missing data are indicated using by symbol -; Chl: chlorophyll or derivative; UC: undetermined carotenoid; Ax: astaxanthin. * Possible fatty acid combination; ^a^ % of total peak area of the extract considering all pigments detected at 480 nm; ^b^ % of total astaxanthin; ^c^ ratios based on the relative peak areas at 480 nm. Absorbance maxima are indicated in bold. *m*/*z*: ratio of an ion’s mass (m) to its formal charge (z); [M + H]^+^: positive ion; [M − H]^−^: negative ion.

**Table 5:** Free astaxanthin equivalent contents measured by HPLC in the red biomasses of the strains 3RB and Lang2 after the saponification of the extracts.

	3RB	Lang2			
		FMT 1	FMT 2	FMT 3	FMTs Mean
Astaxanthin content (mg g^−1^ dw) ^1^	15.92 ± 3.75 ^a^	11.29 ± 3.17 ^ab^	10.23 ± 1.03 ^b^	12.89 ± 0.95 ^a^	11.35 ± 2.09
Astaxanthin productivity (mg dw L^−1^ d^−1^ dw) ^2^	Nd	0.215	0.256	0.284	0.251 ± 0.035 ^1^

^1^ Presented as mean ± standard deviation (n = 3–4); ^2^ Calculated from the results in Table 3. Different letters in the same line indicate significant differences using Tukey’s test (*p* < 0.05). Nd, not determined.

**Table 6:** The molar concentrations (mM) of nitrogen and phosphorus and N/P ratio in the different media used in this study.

Medium	Compound Concentration (mM)		N/P Ratio
	N	P	
BBM/2	1.47	0.86	1.71
BBM	2.94	1.72	1.71
3N-BBM	8.82	1.72	5.13
BG11 0.5	5.88	0.18	33.60
BG11 1.0	11.76	0.18	67.20
BG11 1.5	17.65	0.18	100.86

**Table 7:** A summary of some of the biomass and astaxanthin productivities reported in the literature for different *H. lacustris* strains and culture processes.

*H. lacustris* Strain	PBR Type	Volume (L)	Mode	µ_max_ (d^−1^)	Green Biomass Productivity (g L^−1^ d^−1^)	Red Biomass Productivity (g L^−1^ d^−1^)	Astaxanthin Content (mg g^−1^)	Astaxanthin Productivity (mg L^−1^ d^−1^)	Reference
AQSE002	Tubular PBR/open ponds	25,000	P, O, F	0.13	0.04–0.05	–	28–30	–	[43]
CCAP 34/8	Bubbling column	55	P, O, B	0.50	–	0.06	2.0	0.12 ^a^	[48]
	Tubular airlift PBR	55	P, O, B	0.96	–	0.41	11.0	4.4 ^a^	
CCAP 34/8	Bubbling column	1.8	P, I, C	0.96	0.58	–	–	–	[44]
	Tubular airlift PBR	220	P, O, C	0.48	0.68	–	–	–	
CCAP 34/8	Bubbling column	1.8	P, I, C	–	–	1.9	11	21 ^a^	[55]
CCAP 34/8	Tubular airlift PBR	50	P, O, C	0.60	–	0.6–0.7	6–13	3.5–8.0 ^a^	[47]
CCAP 34/7	Airlift column	30	P, I, B	–	0.03	0.01	27	0.44 ^b^	[54]
K-0084	Bubbling column	0.5	P, I, F	–	0.50	0.21	40	11.5 ^b^	[20]
	Flat panel/tubular PBR	500/2000	P, O, F	0.28	0.37	0.21	38	10.1 ^b,c^	
K-0084	Bubbling column	0.6	P, I/O, B	–	–	0.58	27	17.1 ^d^	[56]
K-0084	Bubbling column	0.6	P, I/O, B	–	–	0.3	38	16 ^d^	[57]
NIES-144	Bubbling column	1	P, I, F	–	0.36	0.14	36	12 ^b^	[45]
NIES-144	Airlift column	1	P, I, F	–	0.40	–	–	18 ^b^	[58]
NIES-144	Flask	1	H, I, B	–	–	–	23.7	10.5 ^d^	[59]
UTEX 16	Bioreactor	2.5	M, I, F	0.58	–	0.14	20–23	3.2 ^a^	[46]
Lang2	Flat panel vertical	3	P, I, F	0.49	0.04	0.013	13	0.28 ^b^	This study

PBR: photobioreactor; P: photoautotrophy; M: mixotrophy; H: heterotrophy; I: indoor; O: outdoor; C: continuous; B: batch; F: fed-batch. ^a^ One-step culture process; ^b^ two-step culture process—the productivity value was calculated based on the total culture time; ^c^ the induction of astaxanthin production was performed outdoors; ^d^ two-step culture process—the productivity value was calculated based only on the time of the red stage.

## Data Availability

The original contributions presented in the study are included in the article, further inquiries can be directed to the corresponding authors.
